# Diet intake and adherence to recommendations in women with gestational diabetes mellitus

**DOI:** 10.1038/s41430-025-01596-z

**Published:** 2025-03-19

**Authors:** Lotta Saros, Tero Vahlberg, Outi Pellonperä, Kristiina Tertti, Kirsi Laitinen

**Affiliations:** 1 Integrative Physiology and Pharmacology Unit, Institute of Biomedicine, University of Turku, 20520 Turku, Finland; 2 Department of Biostatistics, University of Turku and Turku University Hospital, 20520 Turku, Finland; 3 Department of Obstetrics and Gynecology, University of Turku and Turku University Hospital, 20520 Turku, Finland; 4 Nutrition and Food Research Center, University of Turku, 20520 Turku, Finland

**Keywords:** Obesity, Gestational diabetes

## Abstract

**Background/objectives:**

Gestational diabetes mellitus (GDM) is best managed via lifestyle changes. We aimed at investigating to which extent women with GDM adhered to dietary recommendations and to which extent an impact was observed on the glycaemic control compared to women without GDM.

**Subjects/methods:**

Women with overweight/obesity (*n* = 349) were recruited in early pregnancy. GDM was diagnosed with a 2-h oral glucose tolerance test in mid-or-early pregnancy (median 25.9 and 14.7 gestational weeks). Dietary assessments included an index of dietary quality (good ≥ 10 and poor < 10/15 scores) and 3-day food-diaries with nutrient intake calculated and dietary patterns identified. Glucose and insulin concentrations were analysed from blood samples collected in late pregnancy (after GDM diagnosis).

**Results:**

Women with GDM (*n* = 98) followed more often a healthier dietary pattern (62.2%) than women without GDM (49.0%, *p* < 0.05), but no difference in good dietary quality was seen (53% vs. 59.8%, *p* = 0.071). While the majority of women with GDM adhered to some recommendations, 51% to carbohydrate, 54.1% to total fat and 69.4% to sucrose, only 16.3% adhered to the protein and 4.1% to the fibre intake recommendations. Women with GDM had lower adherence to protein, total fat and fibre recommendations but higher adherence to that of sucrose than women without GDM (*p* < 0.05). A good dietary quality was associated with lower insulin and HOMA2-IR values (*p* < 0.05).

**Conclusions:**

Adherence to dietary recommendations, particularly fibre and protein intake, is unsatisfactory in women with GDM. Overall dietary quality is related to better control of glucose metabolism proposing a target for dietary counselling.

## Introduction

Gestational diabetes mellitus (GDM) is a common disorder of glucose metabolism that is detected for the first time in pregnancy; its global prevalence is estimated to be 15.8% [1, 2]. The main risk factors for the disease are overweight and obesity (body mass index, BMI 25–29.9 kg/m^2^ and ≥30 kg/m^2^), and in Finland in 2021, almost 40% of pregnant women were with overweight [3]. GDM predisposes women to complications for both mother and child, including preeclampsia, complications at delivery, inclusive of the need for caesarean section, shoulder dystocia, physical birth trauma and admission to the neonatal intensive care unit, and later in life to an increased risk for type 2 diabetes [4–7]. Children born to women with GDM have an increased risk of suffering health conditions like obesity, impaired glucose tolerance and diabetes [8]. Due to the multiple adverse impacts on health, the management of GDM aims primarily to control glucose metabolism.

Lifestyle modifications, including diet and physical activity are the first steps in the management of GDM. It is estimated that the majority, 70–85%, of the cases can be controlled with these changes alone [9], whilst the rest will need medication with insulin or metformin to keep glucose levels at recommended levels [9]. Dietary counselling focuses on the frequency of eating, limiting the intake of carbohydrates and controlling weight gain by restricting energy intake [2, 10]. An overall good nutritional quality of the consumed foods should be emphasised, focusing on the intake of vegetables, fruits, whole-grains, low-fat dairy-products and fish [10].

Maternal glucose control is largely dependent on adherence to the guidelines set for the management of GDM. However, there is a lack of evidence on how well pregnant women actually adhere to these recommendations. It has been claimed that adherence to lifestyle counselling can be challenging for many reasons e.g. tiredness, insufficient time and lack of knowledge or support [11, 12]. On the other hand, the desire to give birth to a healthy baby has been found to be a major factor motivating pregnant women with overweight or obesity to adopt a healthy lifestyle [12].

Our primary aim was to investigate the adherence to the dietary recommendations and nutrient intake of women with GDM and to compare these to the respective information obtained from the women without GDM. Secondly, we studied separately women with GDM who were treated by diet only and women who required medication to control glucose status. Third, we investigated the association of the diet with glycaemic control in women with or without GDM. We chose to study women with overweight and obesity as they are an at-risk population considering the onset of GDM as well as potential health complications occurring during pregnancy.

## Methods

The data for this study were collected in a longitudinal, mother-child dietary-intervention trial (ClinicalTrials.gov, NCT01922791); its recruitment took place between October 2013 and July 2017, reported in detail previously [13]. The study was carried out according to the guidelines of the Declaration of Helsinki; the study protocol was approved by the Ethics Committee of the Hospital District of Southwest Finland and an informed consent was collected from all the participants.

We recruited healthy pregnant women (*n* = 439) with inclusion criteria of <18 gestational weeks (GW), self-reported pre-pregnancy BMI ≥ 25 kg/m^2^ and singleton pregnancy. GDM was diagnosed in mid-pregnancy (median 25.9, IQR = 25.0–27.4 GW) or in early pregnancy (high-risk women, *n* = 26) (median 14.7, IQR = 12.9–16.0 GW) [14]. Dietary assessments and blood sampling (glucose and insulin concentrations) were conducted at mean 35.2 (SD = 0.97) GW, i.e. after the GDM diagnosis. Gestational weight gain during the whole pregnancy was defined as the last measured weight minus the self-reported pre-pregnancy weight. Women’s clinical characteristics were collected by questionnaires and interviews at baseline. Physical activity was assessed by a questionnaire [15] and a metabolic equivalent index for physical activity (MET-index, h/wk) was calculated and categorised into three groups (light, moderate, vigorous) [16]. No dietary counselling was given during the study visits, but all pregnant women, regardless of the GDM status attend communal maternity clinics monthly, and during the last trimester of pregnancy on a weekly basis. These visits include diet counselling provided by trained public health nurses in addition to maternal and foetal health check-ups. Pregnant women, particularly women with obesity, are advised to follow dietary recommendations: increase their consumption of vegetables, fruits and berries, choose whole grain cereal products, low-fat dairy products and meat products with a preference towards lean meat including poultry and fish and to choose spreads and salad dressing with unsaturated fatty acids.

### GDM diagnosis, blood sampling and analyses

GDM diagnosis was based on a 2-h 75-g oral glucose tolerance test (cut-off values 0 h ≥ 5.3, 1 h ≥ 10.0, 2 h ≥ 8.6 mmol/L) according to the Finnish Current Care Guidelines [14]. The women diagnosed with GDM were treated in maternity clinics, which are part of the Finnish public health system [17]. In Finland, dietary treatment of GDM follows nutritional recommendations for pregnancy with administration of metformin and/or insulin if needed to help control blood glucose-levels [14].

Fasting (>9 h) blood glucose levels were measured by a hexokinase-based enzymatic method and serum insulin by immunoelectrochemiluminometry in a certified laboratory (TYKSLAB, Hospital District of Southwest Finland). Insulin resistance was determined by calculating the HOMA (HOMA2-IR) [18].

### Dietary intake

Dietary data were collected from three-day food diaries (two weekdays and one weekend day). Participants were given oral and written instructions on how to complete the food diary with the accuracy being checked with the help of an illustrated portion booklet. The intake of energy, energy-yielding nutrients, vitamins and minerals was calculated with computerised software AivoDiet 2.0.2.3 (Aivo, Turku, Finland). The intake from food supplements was recorded from food diaries and added to the dietary intake (total intake). Adherence to the dietary recommendations was based on the Finnish recommendations [19] as well as from the 2013 care guidelines for treatment of gestational diabetes as the data were collected at that time. The recommended values are presented in result tables.

Dietary patterns were identified based on the consumption of food groups calculated from food diaries as described previously [20]. Two dietary patterns were identified; these were designated as either healthier or unhealthier patterns. The healthier pattern was characterised by a high consumption of vegetables, rye bread, fruits, nuts, pasta and rice; the unhealthier pattern by high intake of sugary beverages, multigrain/wheat bread, sweets, and pastries.

The quality of the diet was evaluated by a validated index of the diet quality (IDQ) questionnaire [21] which consists of 18 questions investigating the amount and frequency of consumption of certain food products and beverages (whole-grain bread, saturated/unsaturated fatty acids, dairy products, vegetables, fruits, berries, sugar-containing drinks and sweets). Since its maximum points were 15, after scoring the questions, the quality of diet was considered to be good ( ≥ 10/15) or poor (<10/15) [21].

The mean length of the fasting time, i.e. time between the last meal in the evening and the first meal in the morning and the number of meals per day were calculated from the food diaries [20]. Meals were categorised as food-containing meals or beverage meals (beverages without calories were not considered as meals).

### Statistical analyses

The normality of the data was assessed by skewness-values (<1). Mean (standard deviation) were reported for normally distributed variables and median (interquartile range) for values not normally distributed. Independent Samples T-test was used when comparing between two groups for normally distributed variables, otherwise we applied Mann-Whitney U-test. Categorical variables were reported as frequency (%), with Chi Square test or Fisher’s Exact Test being used to detect associations. General linear models were performed to investigate the associations between GDM-status (without vs. with GDM or diet vs. medicine treated GDM), dietary quality (good vs. poor), dietary patterns (healthier vs. unhealthy) and insulin/glucose concentrations or nutrient intake. Logistic regression models were applied to examine if there were associations between GDM-status (without vs. with GDM, diet vs. medicine treated GDM, without GDM vs. diet-treated GDM) and adherence to recommendations, dietary patterns and dietary quality. The analyses were adjusted based on group differences in the participants’ characteristics: (1) women without vs. women with GDM, maternal education, previous GDM diagnosis, pre-pregnancy BMI, (2) without GDM vs. diet-treated GDM, previous GDM diagnosis and pre-pregnancy BMI, (3) dietary quality (good vs. poor), maternal education level and age. In addition, the original trial intervention-groups (fish oil and/or probiotics or placebo) were included in these models. Gestational weight gain was not included in the models to avoid multicollinearity problems as it correlated with pre-pregnancy BMI (*r* = −0.25, *p* < 0.001). GW at delivery was not included as it did not affect the outcomes. Prior to the adjusted analysis, variables that were not normally distributed were natural-log transformed. Statistical analyses were conducted using IBM SPSS 27.0 (SPSS Inc., Chicago, IL, USA). *P*-value < 0.05 was considered statistically significant.

## Results

### Clinical characteristics

Almost every third woman was diagnosed with GDM, with the majority (80%) being treated with diet only, whilst the remaining 20% received additionally either metformin (12%), insulin (3.1%) or both (5.1%). The women with GDM had a higher pre-pregnancy BMI and a lower education level when compared to women without GDM (Table 1). No differences in the clinical characteristics were detected between women treated with diet alone or women supplemented with medicine (Table S1). The clinical characteristics according to women’s dietary quality and dietary pattern are shown in the supplementary table (Table S2).

**Table 1:** Clinical characteristics at baseline of all women and those without and those with GDM.

	All (*n* = 349)	Without GDM (*n* = 251)	With GDM (*n* = 98)	*p*
Mother				
Age (years)	30.7 (4.5)	30.4 (4.5)	31.3 (4.6)	0.097
College/university education	217 (62.7)	164 (66.1)	53 (54.1)	0.048^a^
Primipara	170 (48.7)	124 (49.4)	46 (46.9)	0.721
Previous GDM	30 (8.6)	11 (4.4)	19 (19.4)	<0.001^a^
Smoking during pregnancy	13 (3.8)	9 (3.7)	4 (4.1)	1.000
Smoking before pregnancy	69 (19.9)	54 (21.8)	15 (15.3)	0.232
Pre-pregnancy BMI	28.6 (26.5–31.7)	28.4 (26.3–31.1)	30.0 (27.1–33.5)	0.001^a^
Overweight	216 (61.9)	167 (66.5)	49 (50.0)	
Obesity	133 (38.1)	84 (33.5)	49 (50.0)	0.005
Gestational weight gain, kg	13.1 (6.26)	13.9 (6.13)	11.1 (6.19)	<0.001^a^
Physical activity, MET hours/week	3.0 (0.5–7.5)	3.0 (0.2–7.5)	3.0 (0.5–7.5)	0.581
Child				
Gestational weeks at birth	39.9 (39.0–40.6)	40.1 (39.3–40.9)	39.4 (38.4–40.3)	<0.001^a^
Birth weight, g	3621 (515)	3641 (513)	3571 (518)	0.251
Macrosomia	16 (4.6)	11 (4.4)	5 (5.1)	0.779

Normally distributed variables are presented as means (standard deviation) and non-normally distributed values as medians (interquartile range), categorical variables as frequencies (%). Independent Samples *t*-test, Mann Whitney U-test, Chi squared or Fisher exact test.

^a^Significant value (*p* < 0.05).

BMI body mass index, GDM gestational diabetes mellitus, Overweight BMI 25-29.9 kg/m^2^, Obesity BMI ≥ 30 kg/m^2^.

Baseline: mean 13.9, standard deviation 2.1 gestational weeks.

With GDM treated with diet only or medicine.

### Dietary patterns, dietary quality and meal frequency

A higher proportion of women with GDM, 62.2%, adhered to a healthier dietary pattern in comparison to the women without GDM (49.0%, *p* = 0.032) (Table 2). When one considers the treatment modality for GDM, a higher proportion of women treated with medicine (85%) were following a healthier dietary pattern when compared to women treated with diet only (56.4%, *p* = 0.021) (Table 2). There were no statistically significant differences between women with GDM (59.8%) and women without GDM (53.0%, *p* = 0.280) or between women with GDM treated with diet only (57.1%) and supplemented with medicine (70.0%, *p* = 0.321) with respect to the numbers consuming a good quality diet (Table 2).

**Table 2:** Comparisons of adherence to the dietary patterns and dietary quality between women without GDM and with GDM; between women with GDM treated with diet only and women with GDM treated with medicine (insulin and/or metformin), and between women without GDM and women with GDM treated with diet only.

	*n*	Healthier dietary pattern	Unhealthier dietary pattern	*p*	Adjusted OR (95% CI)	Adjusted p^a^
Without GDM	251	123 (49.0)	128 (51.0)	0.032^b^	1.73 (1.03; 2.89)	0.037^1b^
With GDM	98	61 (62.2)	37 (37.8)			
GDM diet only	78	44 (56.4)	34 (43.6)	0.021^b^	4.49 (1.19; 16.9)	0.026^2b^
GDM medicine	20	17 (85.0)	3 (15.0)			
Without GDM	251	123 (49.0)	128 (51.0)	0.300	1.48 (0.86; 2.53)	0.155^3^
GDM diet only	78	44 (56.4)	34 (43.6)			

	*n*	Good dietary quality	Poor dietary quality	*p*	Adjusted OR (95% CI)	Adjusted p^a^
Without GDM	249	132 (53.0)	117 (47.0)	0.280	1.55 (0.92; 2.62)	0.099^1^
With GDM	97	58 (59.8)	39 (40.2)			
GDM diet only	77	44 (57.1)	33 (42.9)	0.321	2.22 (0.72; 6.91)	0.167^2^
GDM medicine	20	14 (70.0)	6 (30.0)			
Without GDM	249	132 (49.0)	117 (47.0)	0.601	1.32 (0.76; 2.26)	0.322^3^
GDM diet only	77	44 (57.1)	33 (42.9)			

Values are shown as frequencies (%), Fisher exact test.

^a^Logistic regression model, adjusted for ^1^ pre-pregnancy BMI, education level, previous GDM diagnosis and intervention groups, ^2^intervention groups, ^3^previous GDM diagnosis, pre-pregnancy BMI and intervention groups.

OR for healthier dietary pattern and good dietary quality. ^1^Without GDM, ^2^GDM diet only and ^3^Without GDM as reference categories.

Missing data on dietary quality, *n* = 2.

^b^Significant value (*p* < 0.05).

With GDM=treated with diet only or medicine.

CI Confidence interval, GDM Gestational diabetes mellitus, OR Odds ratio.

In women with GDM, the mean fasting time was 11.7 (1.6) hours, which did not differ from that of women without GDM (11.9 (1.9)), *p* = 0.45. The women with GDM had a mean 5.2 (1.0) meals/day, including beverage meals, while the corresponding value in women without GDM was 5.4 (1.1) (*p* = 0.21). The result remained unchanged, when considering only the meals containing food (data not shown).

### Adherence to dietary recommendations and intake of nutrients

#### Energy and energy-yielding nutrients

Approximately half of the women with GDM met the recommendations with respect to the intake of carbohydrate and total fat, even more, 69.4%, for sucrose intake, whilst less than one out of every five met the criterion for adequate protein intake (Table 3). Furthermore, only a small proportion (4.1%) adhered to the recommendation for fibre intake. When investigating whether the adherence to dietary recommendations differed between women with and without GDM (Table 3), we observed that a lower proportion of women with GDM met the recommendations for protein, total fat, and fibre intake than the women without GDM. In contrast, a higher proportion of women with GDM adhered to the sucrose recommendation when compared to women without GDM (Table 3). No differences were detected according to women’s treatment modality for GDM (Table S3). In both groups, the adherence to fibre recommendation was extremely low (3.8% and 5.0% in diet only and medicine groups, respectively).

**Table 3:** Comparison of intakes of energy and energy-yielding nutrients and adherence to diet recommendations between women without GDM and those with GDM.

		Without GDM (*n* = 251)	With GDM (*n* = 98)	*p*	Adjusted p^a^
Energy	MJ	8.5 (2.1)	7.7 (2.0)	0.003^b^	0.014^b^
	*meets rec*.	38 (15.1)	19 (19.4)	0.337	0.871
Carbohydrates	E%	45.4 (5.8)	41.8 (7.6)	<0.001^b^	<0.001^b^
	g	226.0 (62.2)	189.3 (57.1)	<0.001^b^	<0.001^b^
	*meets rec*.	131 (52.2)	50 (51.0)	0.905	0.888
Protein	E%	16.4 (2.9)	17.8 (3.6)	0.001^b^	0.002^b^
	g	80.7 (20.3)	80.0 (23.6)	0.775	0.900
	*meets rec*.	227 (90.4)	16 (16.3)	<0.001^b^	<0.001^b^
Fat					
Total fat	E%	35.8 (6.2)	37.8 (7.0)	0.009^b^	0.027^b^
	g	82.6 (27.0)	80.2 (28.3)	0.460	0.571
	*meets rec*.	174 (69.3)	53 (54.1)	0.009^b^	0.042^b^
SFA	E%	13.3 (3.3)	13.8 (3.3)	0.209	0.366
	g	30.7 (11.1)	29.2 (10.8)	0.239	0.303
MUFA	E%	12.4 (2.6)	12.9 (3.0)	0.109	0.219
	g	26.9 (21.0–34.7)	26.5 (20.1–33.0)	0.197	0.282
PUFA	E%	5.3 (4.4–6.3)	5.4 (4.6–6.7)	0.125	0.203
	g	11.9 (9.1–15.5)	12.3 (8.6–14.8)	0.348	0.472
Sucrose	E%	10.4 (8.0–12.6)	7.8 (5.0–10.4)	<0.001^b^	<0.001^b^
	g	50.3 (35.6–69.4)	34.2 (21.0–52.0)	<0.001^b^	<0.001^b^
	*meets rec*.	110 (43.8)	68 (69.4)	<0.001^b^	0.001^b^
Fibre	g	20.1 (15.5–25.1)	19.1 (15.0–24.6)	0.804	0.521
	*meets rec*.	56 (22.3)	4 (4.1)	<0.001^b^	0.001^b^

Normally distributed variables are presented as means (standard deviation) and non-normally distributed as medians (interquartile range), categorical variables as frequencies (%). Independent Samples t-test, Mann Whitney U-test or Chi squared or Fisher exact test.

^a^General linear model or logistic regression model adjusted for pre-pregnancy BMI, education level, previous GDM diagnosis, intervention groups.

^b^Significant value (*p* < 0.05).

MUFA (g), PUFA (E%, g), sucrose (E%, g), fibre (g) are ln transformed for the adjusted analyses due to their skewed distributions.

With GDM treated with diet only or medicine.

*GDM* Gestational diabetes mellitus, *MUFA* Monounsaturated fatty acid, *PUFA* Polyunsaturated fatty acid, *SFA* Saturated fatty acid.

Recommendations for women without GDM per day: 6.7–7.5 MJ (overweight and obese); 45–60 E% carbohydrates (of which maximum of 10 E% sugars); 10–20 E% protein; 25–40 E% fat. Recommendations for GDM positive women per day: 6.7–7.5 MJ (overweight and obese); 40–50 E% carbohydrates (of which maximum of 10 E% sugars); 20–25 E% protein; 30–40 E% fat.

When we examined nutrient intake, women with GDM had a lower intake of energy, carbohydrates, and sucrose when compared to non-GDM women (Table 3). When the intake was assessed as a percentage of energy intake, it was evident that the women with GDM were consuming higher proportions of fat and protein, but lower proportions of carbohydrates and sucrose (Table 3). We also compared the women with GDM treated with diet alone with women who received additionally medication (Table S3); there were only two differences observed i.e. the women with GDM treated with diet (1) had a lower intake of fibre (non-significant in the adjusted model) and (2) higher intake of sucrose.

#### Vitamins and minerals

A lower proportion of women with GDM met the recommendation for calcium intake (intake from diet and diet+supplements) when compared to women without GDM (non-significant in the adjusted model, Table 4). When investigating the women according to the treatment modality for GDM, the women treated with medication had a higher adherence to the pyridoxine recommendation than women treated with diet alone (non-significant in the adjusted model, Table S4).

**Table 4:** Comparisons of intakes of vitamins and minerals and adherence to recommendations between women without GDM and with GDM.

		Without GDM (*n* = 251)	With GDM all (*n* = 98)	*p*	Adjusted p^a^		Without GDM (*n* = 251)	With GDM (*n* = 98)	*p*	Adjusted p^a^
		Dietary intake					Total intake (diet+food supplements)			
Vitamin A	ug	669 (502–861)	617 (483–879)	0.452	0.898	ug	669 (502–864)	637 (504–885)	0.604	0.730
	ug/MJ	78.1 (60.8–101.9)	85.8 (64.6–112)	0.257	0.137	ug/MJ	78.7 (60.8–102)	86.5 (65.3–116)	0.202	0.086
	*meets rec*.	77 (30.7)	31 (31.6)	0.898	0.660	*meets rec*.	78 (31.1)	32 (32.7)	0.798	0.553
Thiamine	mg	1.2 (1.0–1.6)	1.2 (1.0–1.5)	0.613	0.492	mg	4.2 (1.6–6.1)	4.2 (1.4–6.2)	0.915	0.336
	mg/MJ	0.15 (0.1–0.2)	0.16 (0.1–0.2)	0.022^b^	0.064	mg/MJ	0.47 (0.2–0.7)	0.53 (0.2–0.8)	0.224	0.100
	*meets rec*.	69 (27.5)	24 (24.5)	0.593	0.324	*meets rec*.	197 (78.5)	71 (72.4)	0.259	0.534
Riboflavin	mg	2.0 (0.7)	1.8 (0.6)	0.045^b^	0.082	mg	3.9 (2.4–6.7)	4.0 (2.1–6.8)	0.769	0.619
	mg/MJ	0.24 (0.07)	0.24 (0.07)	0.818	0.844	mg/MJ	0.47 (0.3–0.8)	0.57 (0.3–0.8)	0.410	0.138
	*meets rec*.	173 (68.9)	61 (62.2)	0.255	0.448	*meets rec*.	219 (87.3)	219 (87.3)	0.391	0.917
Niacin	mg	30.7 (8.2)	30.4 (9.4)	0.761	0.646	mg	45.6 (34.0–52.7)	43.7 (33.0–54.1)	0.761	1.00
	mg/MJ	3.7 (0.77)	4.0 (0.98)	0.005^b^	0.017^b^	mg/MJ	5.2 (4.2–6.3)	5.6 (4.4–7.1)	0.038^b^	0.061
	*meets rec*.	243 (96.8)	95 (96.9)	1.000	0.992	*meets rec*.	248 (98.8)	98 (100.0)	0.562	0.996
Pyridoxine	mg	1.8 (1.5–2.3)	1.8 (1.4–2.2)	0.332	0.333	mg	6.4 (2.5–7.1)	6.2 (2.2–7.0)	0.442	0.779
	mg/MJ	0.22 (0.2–0.3)	0.23 (0.2–0.3)	0.054	0.126	mg/MJ	0.67 (0.3–0.9)	0.68 (0.3–0.9)	0.663	0.137
	*meets rec*.	204 (81.3)	75 (76.5)	0.372	0.577	*meets rec*.	234 (93.2)	88 (89.8)	0.274	0.806
Vitamin B12	ug	4.6 (3.4–5.9)	4.8 (3.1–6.0)	0.861	0.762	ug	6.9 (5.3–8.6)	7.0 (4.8–9.0)	0.937	0.512
	ug/MJ	0.55 (0.4–0.7)	0.62 (0.5–0.8)	0.057	0.185	ug/MJ	0.79 (0.6–1.0)	0.90 (0.7–1.2)	0.059	0.169
	*meets rec*.	240 (95.6)	93 (94.9)	0.779	0.712	*meets rec*.	248 (98.8)	97 (99.0)	1.000	0.387
Vitamin C	mg	128 (81.7–174)	109 (68.7–170)	0.078	0.141	mg	221 (148–287)	191 (124–272)	0.281	0.669
	mg/MJ	14.8 (9.9–20.9)	14.9 (9.3–20.7)	0.610	0.817	mg/MJ	25.4 (16.7–33.9)	25.2 (16.8–36.1)	0.743	0.981
	*meets rec*.	181 (72.1)	66 (67.3)	0.432	0.804	*meets rec*.	218 (86.9)	87 (88.8)	0.721	0.244
Vitamin D	ug	7.7 (5.0–11.0)	7.8 (5.1–11.5)	0.833	0.854	ug	19.2 (13.2–25.9)	19.6 (14.4–24.9)	0.725	0.163
	ug/MJ	0.89 (0.6–1.3)	1.01 (0.7–1.4)	0.080	0.114	ug/MJ	2.2 (1.6–3.0)	2.4 (1.7–3.4)	0.171	0.144
	*meets rec*.	77 (30.7)	33 (33.7)	0.609	0.528	*meets rec*.	213 (84.9)	81 (82.7)	0.626	0.618
Vitamin E	mg	10.1 (7.8–12.4)	9.5 (7.8–12.6)	0.415	0.422	mg	18.2 (7.7)	17.7 (7.6)	0.567	0.837
	mg/MJ	1.2 (1.0–1.4)	1.3 (1.1–1.5)	0.117	0.114	mg/MJ	2.2 (0.91)	2.3 (1.0)	0.160	0.046^b^
	*meets rec*.	127 (50.6)	41 (41.8)	0.154	0.366	*meets rec*.	203 (80.9)	75 (76.5)	0.377	0.896
Folate	ug	244 (191–291)	234 (181–286)	0.316	0.822	ug	559 (243)	541 (251)	0.549	0.989
	ug/MJ	29.1 (24.2–34.1)	30.4 (25.4–36.6)	0.067	0.013^b^	ug/MJ	67.7 (32.0)	72.4 (36.1)	0.235	0.119
	*meets rec*.	2 (0.8)	1 (1.0)	1.000	0.809	*meets rec*.	166 (66.1)	63 (64.3)	0.802	0.669
Vitamin K	ug	100 (77.8–131)	92.9 (69.4–119)	0.102	0.264	ug	107 (77.8–132)	93.1 (69.3–124)	0.318	0.469
	ug/MJ	11.7 (9.31–15.5)	12.0 (9.57–14.9)	0.619	0.590	ug/MJ	11.7 (9.31–15.7)	12.1 (9.56–15.7)	0.553	0.513
Calcium	mg	1151 (390)	1053 (416)	0.040^b^	0.095	mg	1218 (441)	1124 (450)	0.079	0.133
	mg/MJ	138 (42.0)	138 (45.8)	0.935	0.932	mg/MJ	145 (47.4)	148 (56.5)	0.640	0.699
	*meets rec*.	183 (72.9)	58 (59.2)	0.015^b^	0.060	*meets rec*.	191 (76.1)	64 (65.3)	0.045^b^	0.195
Magnesium	mg	329 (91.7)	318 (86.1)	0.315	0.715	mg	447 (348–542)	413 (318–539)	0.319	0.915
	mg/MJ	39.1 (33.8–44.0)	41.1 (35.9–47.7)	0.012^b^	0.003^b^	mg/MJ	53.6 (42.4–64.6)	54.1 (45.7–68.0)	0.188	0.182
	*meets rec*.	181 (72.1)	64 (65.3)	0.241	0.595	*meets rec*.	215 (85.7)	82 (83.7)	0.620	0.514
Iron	mg	10.6 (8.8–12.9)	10.9 (8.7–12.8)	0.658	0.669	mg	27.9 (14.9–34.8)	27.4 (11.7–37.5)	0.679	0.801
	mg/MJ	1.3 (1.1–1.5)	1.4 (1.2–1.6)	0.002^b^	<0.001^b^	mg/MJ	3.2 (1.7–4.5)	3.2 (1.7–6.3)	0.525	0.323
Zinc	mg	11.4 (2.9)	11.3 (3.0)	0.806	0.894	mg	20.3 (8.1)	20.2 (8.0)	0.914	0.684
	mg/MJ	1.4 (0.2)	1.5 (0.3)	<0.001^b^	<0.001^b^	mg/MJ	2.5 (1.5–3.1)	2.8 (1.7–3.4)	0.088	0.035^b^
	*meets rec*.	198 (78.9)	76 (77.6)	0.774	0.912	*meets rec*.	223 (88.8)	89 (90.8)	0.701	0.205
Selenium	ug	63.8 (52.4–77.0)	66.6 (49.6–77.2)	0.899	0.955	ug	90.6 (69.3–115)	95.2 (64.5–119)	0.895	0.558
	ug/MJ	7.6 (6.5–8.8)	8.5 (7.0–9.7)	0.001^b^	0.010^b^	ug/MJ	10.5 (8.6–13.0)	11.8 (9.0–15.1)	0.031^b^	0.014^b^
	*meets rec*.	145 (57.8)	61 (62.2)	0.469	0.331	*meets rec*.	212 (84.5)	79 (80.6)	0.424	0.978
Iodine	ug	207 (63.0)	19 (63.7)	0.073	0.271	ug	307.0 (104.9)	288.4 (110.9)	0.144	0.577
	ug/MJ	24.1 (19.8–29.2)	24.9 (21.4–29.2)	0.400	0.389	ug/MJ	37.2 (13.4)	38.3 (14.3)	0.526	0.206
	*meets rec*.	168 (66.9)	55 (56.1)	0.064	0.333	*meets rec*.	218 (86.9)	77 (78.6)	0.069	0.559
Copper	mg	1.2 (1.0–1.4)	1.1 (0.9–1.4)	0.233	0.964	mg	1.4 (1.0–2.0)	1.3 (1.0–1.8)	0.162	0.636
	mg/MJ	0.15 (0.1–0.2)	0.2 (0.1–0.2)	0.045^b^	0.004^b^	mg/MJ	0.16 (0.1–0.2)	0.2 (0.1–0.2)	0.350	0.199
	*meets rec*.	181 (72.1)	70 (71.4)	0.895	0.693	*meets rec*.	194 (77.3)	73 (74.5)	0.577	0.979
Manganese	mg	4.6 (3.6–6.1)	4.2 (3.2–5.6)	0.039^b^	0.255	mg	4.9 (3.6–6.5)	4.6 (3.4–5.9)	0.217	0.850
	mg/MJ	0.55 (0.4–0.7)	0.6 (0.4–0.7)	0.855	0.676	mg/MJ	0.58 (0.4–0.8)	0.55 (0.5–0.8)	0.570	0.182
Chromium	ug	20.3 (15.7–26.3)	16.7 (13.7–21.9)	<0.001^b^	0.009^b^	ug	21.2 (16.4–30.7)	18.1 (14.3–29.6)	0.028^b^	0.686
	ug/MJ	2.4 (1.9–3.0)	2.2 (1.9–2.6)	0.137	0.250	ug/MJ	2.5 (2.0–3.5)	2.4 (2.0–3.6)	0.593	0.407
Potassium	g	3.6 (1.0)	3.4 (0.9)	0.144	0.393					
	g/MJ	0.43 (0.10)	0.5 (0.1)	0.068	0.036^b^					
	*meets rec*.	168 (66.9)	61 (62.2)	0.452	0.824					
Phosphorus	mg	1473 (384)	1415 (393)	0.211	0.474					
	mg/MJ	176 (35.2)	185.6 (37.8)	0.028^b^	0.026^b^					
	*meets rec*.	246 (98.0)	97 (99.0)	1.000	0.340					
Sodium	mg	2659 (762)	2636 (779)	0.795	0.900					
	mg/MJ	318 (68.7)	345 (77.6)	0.001^b^	0.006^b^					
Salt	mg	6779 (1950)	6722 (2016)	0.811	0.881					
	mg/MJ	810 (177)	880 (199)	0.001^b^	0.007^b^					

Normally distributed variables are presented as means (standard deviation) and non-normally distributed as medians (interquartile range), categorical variables as frequencies (%). Independent Samples t-test, Mann Whitney U-test or Fisher exact test.

^a^General linear model or logistic regression model adjusted for pre-pregnancy BMI, education level, previous GDM diagnosis, intervention groups.

^b^Significant value (*p* < 0.05).

Vitamin A (diet, total), thiamine (diet, total), riboflavin (total), niacin (total), pyridoxine (diet, total), vitamin B12 (diet, total), vitamin C (diet, total), vitamin D (diet, total), vitamin E (diet), folate (diet), vitamin K (diet, total), magnesium (diet energy density, total), iron (diet, total), zinc (total energy density), selenium (diet, total), iodine (diet energy density), copper (diet, total), manganese (diet, total), chromium (diet, total) are ln transformed for the adjusted analyses due to their skewed distributions.

With GDM=treated with diet only or medicine.

GDM, Gestational diabetes mellitus.

Meets recommended intake according to Finnish nutritional recommendations of nutrient intakes. Recommendations for pregnant women: 800 RE vitamin A; 1.5 mg thiamine; 1.6 mg riboflavin; 17 NE niacin; 1.4 mg pyridoxine; 2.0 μg vitamin B12; 85 mg vitamin C; 10 μg vitamin D; 10 α-TE vitamin E; 500 μg folate; 900 mg calcium; 280 mg magnesium; 9 mg zinc; 3.1 g potassium; 60 mg selenium; 175 μg iodine; 1.0 mg copper; 700 mg phosphorus.

We detected some differences when comparing the intake of vitamins between women with and without GDM; for example, the intake of thiamine (mg/MJ), niacin (E%), vitamin E (mg/MJ) and folate (ug/MJ) was higher but that of riboflavin (mg) was lower in women with GDM than in the women without GDM (see details in Table 4). In the comparison of the mineral intake of women with GDM vs. without GDM, it seemed that the intake of magnesium (mg/MJ), iron (mg/MJ), zinc (mg/MJ), and selenium (mg/MJ) was higher but the intake of calcium (mg), manganese (mg), and chromium (ug) was lower (see details in Table 4). No differences were evident in the comparison of women treated with diet only and women supplemented with medicine (Table S4).

#### Glycaemic control

When investigating the women’s glycaemic control (mean 35.2 GW), we found that women with GDM who were treated with diet only, had a higher fasting glucose value than women without GDM (*p* < 0.001) (Table 5). We also evaluated whether diet affected the glycaemic control; the women with a good dietary quality in both the women without GDM and the GDM treated with diet only (i.e. those receiving insulin or metformin were excluded from this analysis) had lower insulin and HOMA2-IR values when compared to women with a poor dietary quality (Table 5). Dietary patterns were not related to the glucose metabolism values. As per the diagnosis, women without GDM presented with better glycaemic control compared to women with GDM regardless of the diet quality or diet pattern (Table S5).

**Table 5:** Comparison of glucose metabolism values between women without GDM and women with GDM treated with diet only; between women with a good and a poor dietary quality and between women exhibiting a healthier dietary pattern and those displaying an unhealthier pattern.

	Without GDM *n* = 248	GDM diet *n* = 78	*p*	Adjusted p^a 1^
Fasting glucose	4.5 (0.35)	4.8 (0.39)	<0.001^b^	<0.001^b^
Insulin	15.0 (11.0; 19.0)	17.0 (12.0; 24.0)	0.023^b^	0.097
HOMA2-IR	1.9 (1.4; 2.4)	2.1 (1.5; 3.0)	0.014^b^	0.069

	Good dietary quality *n* = 176	Poor dietary quality *n* = 148	p	Adjusted p^a 2^
Fasting glucose	4.5 (0.39)	4.6 (0.37)	0.055	0.116
Insulin	14.0 (11.0; 19.0)	16.0 (12.0; 23.8)	0.008^b^	0.016^b^
HOMA2-IR	1.8 (1.4; 2.4)	2.0 (1.5; 3.0)	0.007^b^	0.017^b^

	Healthier dietary pattern *n* = 167	Unhealthier dietary pattern *n* = 159	*p*	Adjusted p^a 3^
Fasting glucose	4.6 (0.41)	4.6 (0.35)	0.379	0.561
Insulin	15.0 (11.0; 21.0)	15.0 (11.0; 20.0)	0.889	0.639
HOMA2-IR	1.9 (1.4; 2.6)	1.9 (1.4; 2.5)	0.914	0.676

Numbers are shown as mean (standard deviation) or median (interquartile range). Independent Samples t-test or Mann Whitney U-test.

^a^General linear model, adjusted for ^1^pre-pregnancy BMI, previous GDM diagnosis, intervention groups, ^2^education level, age and intervention groups, ^3^intervention groups.

^b^Significant value (*p* < 0.05).

Missing data on dietary quality, *n* = 2.

Insulin and HOMA2-IR values are ln transformed in the adjusted analyses due to their skewed distributions.

*GDM* Gestational diabetes mellitus.

Women with GDM treated with medicine are excluded from the analyses.

## Discussion

In this clinical study, we found that there is room for improvements in the dietary intake of all pregnant women but especially women with GDM; the adherence to the fibre recommendation was at a low level, but also more attention should be paid to the adherence to the carbohydrate, total fat, protein, and energy recommendations. However, when assessing the adherence to dietary pattern, most women with GDM did consume a healthy diet, in fact even more frequently than non-GDM women. A good overall dietary quality was related with better glycaemic control, namely lower insulin and HOMA2-IR values, and this highlights the opportunity for improving their health by dietary therapy.

We observed that the intake of carbohydrates as a percentage of energy was too low compared to dietary recommendations in almost half of the women with GDM. Furthermore, the adherence to the fibre recommendation in these women was at a low level, as less than 5% met the recommended criterion. It is likely that when the women with GDM limit their carbohydrate intake, fibre intake dramatically decreases at the same time. This is worrying as fibre has an important role in glycaemic control [22]. It is noteworthy that at the time the study was conducted the dietary recommendation for women with GDM differed from recommendation for women without GDM such that on average less energy should be derived from carbohydrates (40–50 E% vs. 45–60 E%). The new recommendations were issued in 2024, and now women with GDM are advised to follow essentially the general dietary recommendations for pregnant women (including similar proportions of the energy yielding nutrients) [23]. Clearly, the emphasis should be placed on motivating the women to undertake these lifestyle changes.

We also observed that approximately half of the women did not meet the recommendation for total fat intake and even more (about 84%) for protein intake. As fat is an energy-dense nutrient, it is important to guide women but especially women with overweight or obesity, to use low-fat products as it may affect the weight-gain during pregnancy [24, 25]. An excess weight-gain in turn has been shown to contribute to detrimental health effects for both the mother and the foetus [26, 27]. Considering diet quality or diet pattern we did not observe differences between women with overweight or obesity.

The adherence of women with GDM to the dietary recommendations has been investigated in only a few studies; in one publication from New Zealand, women with GDM (*n* = 313) [28] exhibited poor adherence to the recommendations regarding the intake of saturated fatty acids and whole-grains i.e. similar findings as described here [25]. Another study (*n* = 239, South-Africa) found that only 19.1% of women with GDM received enough fibre from the diet, although that proportion was still higher than in our study [29]. Evidently, more investigations are needed to assess how well women with GDM adhere to dietary recommendations. We also examined whether the dietary intake would be better in women with GDM than in women without GDM: women with GDM had a lower adherence to the protein, fat, and fibre recommendations but a higher adherence to the recommendation for sucrose. A restriction of the consumption of carbohydrates, especially sugary products is a well-known way to manage blood glucose levels, which could explain the relatively good compliance with the sucrose recommendation (69.4%). Unfortunately, the downside is that the restriction of carbohydrate intake typically resulted in a reduced fibre intake. In summary, there is a need to reassess the quality of carbohydrate sources in the diet and how this affects the fibre intake during dietary counselling of these women in maternal health clinics.

Here, 62.2% of all women with GDM and 85% of women treated with medicine followed a healthy dietary pattern. Our findings suggest that dietary counselling in the maternal health clinics to women with GDM, and particularly women being treated with some form of medication is effective to some extent. We have also demonstrated previously that the women who did not develop GDM had a higher adherence to a healthier dietary pattern in early pregnancy when compared to women who developed GDM [20]. On the other hand, the diet of almost every second woman with GDM was not considered to be “healthy”, and not nearly all women met the dietary recommendations. The composition of the diet is known to affect glucose metabolism, and in fact we observed that women with a good dietary quality, defined by an index for dietary quality [21], had better insulin and HOMA2-IR values when compared to women whose dietary quality was rated as poor. This is in line with an American study (*n* = 1220) reporting better glycaemic control in women with GDM who had a higher dietary quality measured by the Healthy Eating Index 2010 [30]. This overall diet approach can form a foundation for dietary counselling as good dietary quality is defined as including a high consumption of whole-grains, vegetables, fruits and berries, low-fat dairy products, and unsaturated fatty acids. Interestingly, our results indicate that the adherence of women with GDM to dietary guidelines was not sufficient to modulate blood glucose values to the same level as observed in women without GDM, probably also reflecting pancreatic insufficiency and intensified insulin resistance in women with GDM.

The strength of our study is its prospective design. We assessed each mother’s dietary intake in detail (intake of energy, energy-yielding nutrients, vitamins and minerals, eating frequency, dietary patterns) from food diaries and also used a validated index to assess the dietary quality [21]. We took possible confounding factors into consideration in our statistical analyses. One limitation is the small number of GDM women treated with some form of medicine (*n* = 20). Another limitation is the usage of dietary patterns, which are data-based and thus depend on the study population. Further, as diet was assessed after the GDM diagnosis, it is possible that the women misreported the intake either consciously or unconsciously.

Evidently, not all women with GDM are adhering to dietary recommendations and thus dietary counselling of this group of women should be intensified especially with respect to the intake of fibre, protein and fat, and this should be emphasised by clinicians. There seems to be a need to develop novel means for dietary counselling, targeted particularly to women at high risk for pregnancy complications e.g. due to overweight/obesity, to improve dietary intake and consequently glycaemic control.

## Supplementary information

Supplement tables

## Data Availability

The datasets analysed during the current study are not publicly available as these datasets contain information that could compromise the privacy and consent from the participants.
