# Supplementary material for: Diet intake and adherence to recommendations in women with gestational diabetes mellitus
Source: Eur J Clin Nutr. 2025 Mar 19;79(7):676–84. doi: 10.1038/s41430-025-01596-z (PMC12274126; doi:10.1038/s41430-025-01596-z)
Supplement: Supplementary file 1 — Supplement tables [file 41430_2025_1596_MOESM1_ESM.pdf]

Diet intake and adherence to recommendations in women with gestational diabetes mellitus

Authors

Lotta Saros, Tero Vahlberg, Outi Pellonperä, Kristiina Tertti, Kirsi Laitinen

Corresponding author

Lotta Saros, loevpa@utu.fi

Integrative Physiology and Pharmacology Unit, Institute of Biomedicine, University of Turku, 20520 Turku, Finland

Table S1 Comparisons of the clinical characteristics at baseline between women without GDM and women with GDM treated with diet only and between women with GDM treated with diet only and women with medication (insulin and/or metformin)

	Without GDM (n=251)	GDM diet (n=78)	p	GDM diet (n=78)	GDM medicine (n=20)	p
**Mother**	n=251	n=78		n=78	n=20	
Age, years	30.4 (4.5)	31.2 (4.8)	0.182	31.2 (4.8)	31.7 (4.0)	0.653
College/university education	164 (66.1)	42 (53.8)	0.059	42 (53.8)	11 (55.0)	1.000
Primipara	124 (49.4)	36 (46.2)	0.697	36 (46.2)	10 (50.0)	0.806
Previous GDM	11 (4.4)	12 (15.4)	0.004*	12 (15.4)	7 (35.0)	0.061
Smoking during pregnancy	9 (3.7)	4 (5.1)	0.521	4 (5.1)	0 (0.0)	0.579
Smoking before pregnancy	54 (21.8)	12 (15.4)	0.260	12 (15.4)	3 (15.0)	1.000
Pre-pregnancy BMI	28.4 (26.3-31.1)	29.7 (26.7-33.5)	0.027*	29.7 (26.7-33.5)	30.6 (28.8-33.3)	0.169
Overweight<br>Obesity	167 (66.5)<br>84 (33.5)	40 (51.3)<br>38 (48.7)	0.016	40 (51.3)<br>38 (48.7)	9 (45.0)<br>11 (55.0)	0.803
Gestational weight gain, kg	13.4 (6.13)	10.9 (6.26)	<0.001*	10.9 (6.26)	11.8 (5.99)	0.568
Physical activity, MET hours/week	3.0 (0.2-7.5)	3.0 (0.2-7.5)	0.920	3.0 (0.2-7.5)	0.6 (0.0-3.0)	0.181
**Child**						
Gestational weeks at birth	40.1 (39.3-40.9)	39.4 (38.7-40.5)	0.003*	39.4 (38.7-40.5)	39.2 (38.2-40.0)	0.126
Birth weight, g	3641 (513)	3546 (531)	0.155	3546 (531)	3668 (466)	0.347
Macrosomia	11 (4.4)	3 (3.8)	1.00	3 (3.8)	2 (10)	0.269

Normally distributed variables are presented as means (standard deviation) and non-normally distributed as medians (interquartile range), categorical variables as frequencies (%). Independent Samples t-test, Mann Whitney U-test, Chi squared or Fisher exact test.

* Significant value ($p<0.05$)

BMI body mass index, GDM gestational diabetes mellitus, Overweight BMI 25-29.9 kg/m^2, Obesity BMI ≥ 30 kg/m^2

Baseline: mean 13.9, standard deviation 2.1 gestational weeks.

Table S2 Comparisons of the clinical characteristics at baseline between women with good dietary quality and poor dietary quality and between women with a healthier dietary pattern and an unhealthier dietary pattern.

	Good dietary quality (n=190)	Poor dietary quality (n=156)	p	Healthier dietary pattern (n=184)	Unhealthier dietary pattern (n=165)	p
**Mother**						
Age, years	31.4 (4.5)	29.8 (4.5)	<0.001	30.6 (4.7)	30.7 (4.4)	0.81
College/university education	135 (71.1)	81 (52.9)	<0.001	106 (57.9)	111 (68.1)	0.051
Primipara	94 (49.5)	75 (48.1)	0.83	92 (50.0)	78 (47.3)	0.61
Previous GDM	17 (8.9)	13 (8.3)	0.84	17 (9.2)	13 (7.9)	0.65
GDM in current pregnancy	58 (30.5)	39 (25.0)	0.26	61 (33.2)	37 (22.4)	0.03
Smoking during pregnancy	4 (2.1)	9 (5.9)	0.07	8 (4.4)	5 (3.1)	0.53
Smoking before pregnancy	32 (16.8)	37 (24.2)	0.09	40 (21.9)	29 (17.8)	0.35
Pre-pregnancy BMI	28.6 (26.3; 31.2)	28.6 (26.5; 32.3)	0.58	28.6 (26.5; 31.4)	28.8 (26.4; 31.8)	1.0
Overweight<br>Obesity	118 (62.1)<br>72 (37.9)	97 (62.2)<br>59 (37.8)	1.0	113 (61.4)<br>71 (38.6)	103 (62.4)<br>62 (37.6)	0.85
Gestational weight gain, kg	13.2 (5.9)	13.1 (6.7)	0.89	13.5 (6.4)	12.7 (6.1)	0.23
Physical activity, MET hours/week	3.0 (0.5; 7.5)	3.0 (0.5; 7.5)	0.18	3.0 (0.35; 7.5)	3.0 (0.5; 7.5)	0.64
**Child**						
Gestational weeks at birth	39.7 (1.4)	39.8 (1.3)	0.29	39.7 (1.5)	40.0 (1.3)	0.18
Birth weight, g	3628 (490)	3619 (544)	0.86	3645 (510)	3594 (521)	0.36
Macrosomia	7 (3.7)	9 (5.8)	0.36	11 (6.0)	5 (3.0)	0.19

Normally distributed variables are presented as means (standard deviation) and non-normally distributed as medians (interquartile range), categorical variables as frequencies (%). Independent Samples t-test, Mann Whitney U-test, Chi squared or Fisher exact test.

* Significant value ($p<0.05$)

Missing data on dietary quality, n=2

BMI body mass index, GDM gestational diabetes mellitus, Overweight BMI 25-29.9 kg/m^2, Obesity BMI ≥ 30 kg/m^2

Baseline: mean 13.9, standard deviation 2.1 gestational weeks.

GDM=treated with diet only or medicine.

Table S3 Comparison of intakes of energy and energy-yielding nutrients and adherence to recommendations between women with GDM treated with diet only or medication (insulin and/or metformin)

		GDM diet (n=78)	GDM medicine (n=20)	p	Adjusted p †
Energy	MJ	7.7 (2.0)	7.8 (2.2)	0.799	0.875
	meets rec.	15 (19.2)	4 (20.0)	1.000	0.846
Carbohydrates	E%	43.3 (37.4-47.2)	40.9 (37.8-44.0)	0.136	0.074
	g	192.2 (56.2)	178.0 (60.7)	0.326	0.314
	meets rec.	39 (50.0)	11 (55.0)	0.804	0.696
Protein	E%	17.5 (3.5)	18.9 (3.5)	0.110	0.066
	g	78.6 (23.6)	85.5 (23.5)	0.245	0.227
	meets rec.	12 (15.4)	4 (20.0)	0.735	0.507
Fat					
Total fat	E%	37.5 (6.6)	39.2 (8.5)	0.316	0.420
	g	78.9 (26.9)	85.1 (33.2)	0.383	0.488
	meets rec.	45 (57.7)	8 (40.0)	0.210	0.219
SFA	E%	13.7 (3.3)	14.3 (3.4)	0.531	0.607
	g	28.8 (10.7)	30.6 (11.6)	0.515	0.634
MUFA	E%	12.9 (2.7)	13.1 (3.8)	0.762	0.866
	g	27.1 (10.0)	28.7 (12.6)	0.557	0.688
PUFA	E%	5.8 (1.6)	5.9 (1.8)	0.821	0.947
	g	11.5 (8.7-14.6)	13.5 (7.2-16.6)	0.395	0.862
Sucrose	E%	8.5 (5.9-11.1)	5.9 (4.1-8.3)	0.021*	0.035*
	g	37.2 (22.5-53.1)	22.5 (15.0-37.6)	0.035*	0.049*
	meets rec.	51 (65.4)	17 (85.0)	0.108	0.139
Fibre	g	18.2 (14.9-23.4)	22.1 (18.7-25.9)	0.042*	0.205
	meets rec.	3 (3.8)	1 (5.0)	1.00	0.617

Normally distributed variables are presented as means (standard deviation) and non-normally distributed as medians (interquartile range). Independent Samples t-test, Mann Whitney U-test or Chi squared or Fisher exact test.

† General linear model or logistic regression model adjusted for the intervention groups.

* Significant value ($p<0.05$)

Carbohydrates (E%), PUFA (g), sucrose (E%, g), fibre (g) are ln transformed in the adjusted analyses due to their skewed distributions.

GDM, Gestational diabetes mellitus; MUFA, Monounsaturated fatty acid; PUFA, Polyunsaturated fatty acid; SFA, Saturated fatty acid.

Recommendations for women with GDM per day: 6.7-7.5 MJ (overweight and obese); 40-50 E% carbohydrates (of which maximum of 10 E% sugars); 20-25 E% protein; 30-40 E% fat.

Table S4. Comparison of intakes of vitamins and minerals and adherence to recommendations between women with GDM treated with diet only or medicine (insulin and/or metformin)

		GDM diet (n=78)	GDM medicine (n=20)	p	Adjusted p †		GDM diet (n=78)	GDM medicine (n=20)	p	Adjusted p †
		Dietary intake					Total intake (diet+food supplements)			
Vitamin A	ug	602 (476-866)	654.0 (512-956)	0.393	0.427	ug	626 (491-867)	654 (512-956)	0.470	0.504
	ug/MJ	82.0 (63.2-110)	95.2 (68.8-124)	0.319	0.359	ug/MJ	82.1 (64.2-116)	95.2 (68.8-124)	0.397	0.439
	meets rec.	22 (28.2)	9 (45.0)	0.181	0.107	*meets rec.*	23 (29.5)	9 (45.0)	0.195	0.144
Thiamine	mg	1.3 (0.4)	1.4 (0.5)	0.201	0.235	mg	4.2 (1.4-6.2)	4.2 (1.8-6.4)	0.515	0.559
	mg/MJ	0.2 (0.0)	0.2 (0.0)	0.194	0.196	mg/MJ	0.5 (0.2-0.8)	0.6 (0.2-0.8)	0.454	0.529
	meets rec.	16 (20.5)	8 (40.0)	0.085	0.094	*meets rec.*	54 (69.2)	17 (85.0)	0.261	0.130
Riboflavin	mg	1.8 (0.6)	1.8 (0.7)	0.954	0.937	mg	4.2 (1.8-6.8)	3.7 (2.4-6.8)	0.765	0.718
	mg/MJ	0.2 (0.1)	0.2 (0.1)	0.867	0.792	mg/MJ	0.6 (0.3-0.9)	0.5 (0.3-0.8)	0.762	0.682
	meets rec.	47 (60.3)	14 (70.0)	0.606	0.456	*meets rec.*	63 (80.8)	19 (95.0)	0.180	0.137
Niacin	mg	29.7 (9.2)	33.0 (10.3)	0.172	0.167	mg	43.4 (16.6)	46.9 (13.2)	0.386	0.352
	mg/MJ	3.9 (1.0)	4.3 (1.0)	0.131	0.086	mg/MJ	5.7 (2.1)	6.3 (1.9)	0.317	0.233
	meets rec.	76 (97.4)	19 (95.0)	0.500	0.490	*meets rec.*	78 (100)	20 (100)	-	-
Pyridoxine	mg	1.8 (0.6)	2.0 (0.5)	0.329	0.421	mg	6.1 (2.1-7.0)	6.5 (3.3-7.0)	0.614	0.457
	mg/MJ	0.2 (0.2-0.3)	0.2 (0.2-0.3)	0.332	0.199	mg/MJ	0.7 (0.3-0.9)	0.7 (0.4-0.8)	0.630	0.428
	meets rec.	56 (71.8)	19 (95.0)	0.037*	0.070	*meets rec.*	68 (87.2)	20 (100)	0.206	0.998
Vitamin B12	ug	4.8 (3.0-6.0)	5.1 (3.1-6.4)	0.509	0.549	ug	7.0 (4.6-9.1)	7.3 (5.4-9.0)	0.571	0.805
	ug/MJ	0.6 (0.5-0.8)	0.6 (0.5-0.7)	0.672	0.436	ug/MJ	0.9 (0.6-1.2)	1.0 (0.7-1.2)	0.309	0.762
	meets rec.	73 (93.6)	20 (100)	0.580	0.998	*meets rec.*	77 (98.7)	20 (100)	1.000	0.998
Vitamin C	mg	124 (71.2)	116 (49.4)	0.657	0.516	mg	189.5 (124-297)	202.2 (126.3-262)	0.950	0.991
	mg/MJ	14.4 (9.3-20.7)	15.2 (9.9-20.7)	0.944	0.936	mg/MJ	25.7 (17.1-36.7)	24.6 (15.6-34.0)	0.775	0.972
	meets rec.	53 (67.9)	13 (65.0)	0.795	0.754	*meets rec.*	70 (89.7)	17 (85.0)	0.691	0.520
Vitamin D	ug	7.2 (4.6-11.5)	8.2 (6.6-11.7)	0.573	0.671	ug	19.5 (13.6-25.3)	21.6 (17.7-23.8)	0.435	0.462
	ug/MJ	1.0 (0.7-1.4)	1.0 (0.8-1.4)	0.771	0.572	ug/MJ	2.4 (1.7-3.4)	2.5 (1.9-3.9)	0.630	0.436
	meets rec.	25 (32.1)	8 (40.0)	0.598	0.434	*meets rec.*	63 (80.8)	18 (90.0)	0.511	0.338
Vitamin E	mg	10.1 (3.6)	10.8 (4.3)	0.474	0.620	mg	17.5 (7.7)	18.5 (7.1)	0.601	0.571
	mg/MJ	1.3 (0.4)	1.4 (0.4)	0.472	0.732	mg/MJ	2.3 (1.0)	2.5 (1.1)	0.449	0.375
	meets rec.	32 (41.0)	9 (45.0)	0.802	0.936	*meets rec.*	58 (74.4)	17 (85.0)	0.389	0.263
Folate	ug	231.3 (180-283)	249.5 (186-288)	0.497	0.711	ug	520.9 (250.9)	618.2 (240.3)	0.122	0.100
	ug/MJ	30.1 (24.9-37.0)	32.5 (26.5-35.6)	0.555	0.582	ug/MJ	69.2 (34.1)	84.7 (41.7)	0.089	0.061
	meets rec.	1 (1.3)	0.0 (0.0)	1.00	0.998	*meets rec.*	47 (60.3)	16 (80.0)	0.122	0.118

Vitamin K	ug	89.1 (68.6-119)	105.7 (83.2-132)	0.157	0.272	ug	88.9 (68.4-121)	106 (83.2-132)	0.227	0.347
	ug/MJ	11.6 (9.4-14.8)	13.1 (10.9-17.1)	0.234	0.208	ug/MJ	11.6 (9.4-15.7)	13.1 (10.9-17.1)	0.293	0.276
Calcium	mg	1063 (412)	1014 (437)	0.640	0.708	mg	111 (448)	1137 (471)	0.889	0.819
	mg/MJ	139 (44.8)	134 (50.5)	0.636	0.773	mg/MJ	136.8 (113-182)	141 (86.3-192)	0.782	0.812
	meets rec.	48 (61.5)	10 (10)	0.446	0.425	*meets rec.*	51 (65.4)	13 (65.0)	1.000	0.921
Magnesium	mg	315.7 (84.9)	325.2 (92.5)	0.664	0.633	mg	438 (163)	466.4 (182.8)	0.507	0.542
	mg/MJ	40.8 (35.6-48.1)	41.6 (38.2-45.7)	0.738	0.616	mg/MJ	54.1 (46.3-68.0)	57.4 (41.6-78.7)	0.649	0.168
	meets rec.	49 (62.9)	15 (75.0)	0.431	0.332	*meets rec.*	64 (82.1)	18 (90.0)	0.513	0.327
Iron	mg	10.1 (8.7-11.9)	12.3 (9.3-13.7)	0.060	0.228	mg	25.9 (11.0-48.2)	30.5 (20.1-37.6)	0.227	0.542
	mg/MJ	1.4 (1.2-1.6)	1.6 (1.3-1.8)	0.064	0.094	mg/MJ	3.2 (1.6-6.3)	3.4 (2.4-6.6)	0.327	0.516
Zinc	mg	11.2 (9.3-12.5)	10.9 (8.7-14.5)	0.721	0.776	mg	20.1 (8.1)	20.3 (8.0)	0.948	0.849
	mg/MJ	1.5 (0.3)	1.5 (0.3)	0.682	0.543	mg/MJ	2.8 (1.7-3.4)	2.6 (1.6-3.3)	0.865	0.793
	meets rec.	62 (79.5)	14 (70.0)	0.378	0.254	*meets rec.*	71 (91.0)	18 (90.0)	1.000	0.845
Selenium	ug	63.3 (48.4-76.8)	69.5 (60.8-87.9)	0.195	0.179	ug	95.2 (61.4-114.9)	95.4 (70.3-129.4)	0.218	0.170
	ug/MJ	8.5 (6.9-9.7)	9.4 (7.9-9.8)	0.141	0.066	ug/MJ	11.4 (8.8-14.9)	13.7 (10.1-16.4)	0.101	0.131
	meets rec.	46 (59.0)	15 (75.0)	0.209	0.273	*meets rec.*	60 (76.9)	19 (95.0)	0.110	0.088
Iodine	ug	193.1 (64.0)	194.0 (64.1)	0.957	0.974	ug	285.5 (115.2)	299.6 (94.4)	0.615	0.570
	ug/MJ	25.2 (6.3)	25.3 (6.9)	0.961	0.949	ug/MJ	37.7 (14.4)	40.3 (13.9)	0.476	0.388
	meets rec.	45 (57.7)	10 (50.0)	0.617	0.434	*meets rec.*	59 (75.9)	18 (90.0)	0.227	0.182
Copper	mg	1.1 (0.9-1.4)	1.3 (1.0-1.4)	0.248	0.547	mg	1.2 (1.0-1.8)	1.4 (1.1-2.0)	0.250	0.400
	mg/MJ	0.2 (0.1-0.2)	0.2 (0.1-0.2)	0.724	0.362	mg/MJ	0.2 (0.1-0.2)	0.2 (0.1-0.2)	0.568	0.302
	meets rec.	54 (69.2)	16 (80.0)	0.416	0.481	*meets rec.*	56 (71.8)	17 (85.0)	0.267	0.298
Manganese	mg	4.0 (3.0-5.2)	5.5 (3.3-6.6)	0.066	0.074	mg	4.4 (3.4-5.6)	5.6 (3.4-7.1)	0.127	0.200
	mg/MJ	0.5 (0.4-0.7)	0.6 (0.5-0.8)	0.153	0.052	mg/MJ	0.6 (0.4-0.8)	0.7 (0.5-0.8)	0.285	0.168
Chromium	ug	16.3 (13.7-23.0)	16.9 (13.9-18.6)	0.785	0.184	ug	18.6 (14.3-29.9)	17.0 (13.9-22.0)	0.553	0.187
	ug/MJ	2.3 (2.0-2.8)	2.1 (1.9-2.5)	0.192	0.124	ug/MJ	2.4 (2.0-3.6)	2.1 (1.9-3.5)	0.281	0.194
Potassium	g	3.4 (0.9)	3.5 (0.8)	0.787	0.787					
	g/MJ	0.5 (0.1)	0.5 (0.1)	0.737	0.618					
	meets rec.	47 (60.3)	14 (70.0)	0.606	0.363					
Phosphorus	mg	1412 (396)	1429 (390)	0.859	0.782					
	mg/MJ	186 (38.9)	186.4 (34.3)	0.924	0.712					
	meets rec.	78 (100)	19 (95.0)	0.204	0.996					
Sodium	mg	2617 (724)	2708 (981)	0.702	0.850					
	mg/MJ	346 (79.4)	342.8 (71.8)	0.879	0.680					
Salt	mg	6667 (1880)	6940 (2523)	0.654	0.790					
	mg/MJ	880 (205)	879 (180)	0.972	0.770					

Normally distributed variables are presented as means (standard deviation) and non-normally distributed as medians (interquartile range), categorical variables as frequencies (%). Independent Samples t-test, Mann Whitney U-test or Fisher exact test.

† General linear model or logistic regression model adjusted for intervention groups.

* Significant value ($p<0.05$)

Vitamin A (diet, total), thiamine (total), riboflavin (total), pyridoxine (diet energy density, total), vitamin B12 (diet, total), vitamin C (diet energy density, total), vitamin D (diet, total), folate (diet), vitamin K (diet, total), calcium (total energy density), magnesium (diet and total energy density), iron (diet, total), zinc (diet energy density, total absolute intake), selenium (diet, total), copper (diet, total), manganese (diet, total), chromium (diet, total) are ln transformed in the adjusted analyses due to their skewed distributions.

GDM, Gestational diabetes mellitus.

Meets recommended intake according to Finnish nutritional recommendations of nutrient intakes.
Recommendations for pregnant women: 800 RE vitamin A; 1.5 mg thiamine; 1.6 mg riboflavin; 17 NE niacin; 1.4 mg pyridoxine; 2.0 µg vitamin B12; 85 mg vitamin C; 10 µg vitamin D; 10 α-TE vitamin E; 500 µg folate; 900 mg calcium; 280 mg magnesium; 9 mg zinc; 3.1 g potassium; 60ug selenium; 175 µg iodine; 1.0 mg copper; 700 mg phosphorus.

Table S5 Comparison of glucose metabolism values between women with and without GDM (treated with diet only) 1) with a good or poor dietary quality or 2) with a healthier or an unhealthier dietary pattern.

	**Without GDM + Good dietary quality n=132**	**Without GDM + Poor dietary quality n=115**	**With GDM + Good dietary quality n=44**	**With GDM + Poor dietary quality n=33**	**p**	**Adjusted p †[1]**
Fasting glucose	4.5 (0.36)	4.6 (0.33)	4.8 (0.38)	4.8 (0.41)	<0.001*	<0.001*[a]
Insulin	14.0 (11.0; 18.0)	16.0 (12.0; 21.0)	17.0 (12.0; 22.0)	17.0 (12.5; 29.0)	0.004*	0.02*[b]
HOMA2-IR	1.8 (1.3; 2.3)	2.0 (1.5; 2.6)	2.1 (1.5; 2.9)	2.2 (1.6; 3.6)	0.002*	0.01*[c]
	**Without GDM + Healthier dietary pattern n=123**	**Without GDM + Unhealthier dietary pattern n=125**	**With GDM + Healthier dietary pattern n=44**	**With GDM + Unhealthier dietary pattern n=34**	**p**	**Adjusted p †[2]**
Fasting glucose	4.5 (0.37)	4.5 (0.32)	4.8 (0.40)	4.8 (0.38)	<0.001*	<0.001*[d]
Insulin	15.0 (11.0; 19.0)	15.0 (11.0; 19.0)	17.0 (11.0; 24.8)	16.5 (12.8; 24.0)	0.16	0.39
HOMA2-IR	1.9 (1.4; 2.4)	1.9 (1.4; 2.4)	2.1 (1.4; 3.1)	2.1 (1.7; 3.0)	0.11	0.32

Numbers are shown as mean (standard deviation) or median (interquartile range). One-Way ANOVA or Kruskal-Wallis Test.

† General linear model, adjusted for [1] pre-pregnancy BMI, previous GDM diagnosis, education level, age, and intervention groups, [2] pre-pregnancy BMI, previous GDM diagnosis, and intervention groups. Insulin and HOMA2-IR values are ln transformed in the adjusted analyses due to their skewed distributions.

* Significant value (p<0.05)

GDM, Gestational diabetes mellitus.

Women with GDM treated with medicine are excluded from the analyses.

Significant differences after Bonferroni adjustment for multiple comparisons:

[a] Without GDM+Poor dietary quality vs With GDM+Poor dietary quality, p=0.03

Without GDM+Poor dietary quality vs With GDM+Good dietary quality, p=0.003

Without GDM+Good dietary quality vs With GDM+Poor dietary quality, p<0.001

Without GDM+Good dietary quality vs With GDM+Good dietary quality, p<0.001

[b] Without GDM+Good dietary quality vs With GDM+Poor dietary quality, p=0.02

[c] Without GDM+Good dietary quality vs With GDM+Poor dietary quality, p=0.01

[d] Without GDM+Healthier dietary pattern vs With GDM+Healthier dietary pattern, p<0.001

Without GDM+Healthier dietary pattern vs With GDM+Unhealthier dietary pattern, p<0.001

Without GDM+Unhealthier dietary pattern vs With GDM+Healthier dietary pattern, p<0.001
